# Household costs of illness during different phases of tuberculosis treatment in Central Asia: a patient survey in Tajikistan

**DOI:** 10.1186/1471-2458-10-18

**Published:** 2010-01-18

**Authors:** Raffael Ayé, Kaspar Wyss, Hanifa Abdualimova, Sadullo Saidaliev

**Affiliations:** 1 Swiss Tropical Institute, Swiss Centre for International Health, Socinstr. 57, 4002 Basel, Switzerland; 2 Project Sino, Rudaki Prospekt Proyezd 5, Dom 1, Dushanbe, Tajikistan; 3 Republican Centre for Tuberculosis Control, Bukhoro street 53, Dushanbe, Tajikistan

## Abstract

**Background:**

Illness-related costs incurred by patients constitute a severe economic burden for households especially in low-income countries. High household costs of illness lead to impoverishment; they impair affordability and equitable access to health care and consequently hamper tuberculosis (TB) control. So far, no study has investigated patient costs of TB in the former Soviet Union.

**Methods:**

All adult new pulmonary TB cases enrolled into the DOTS program in 12 study districts during the study period were enrolled. Medical and non-medical expenditure as well as loss of income were quantified in two interviews covering separate time periods. Costs of different items were summed up to calculate total costs. For missing values, multiple imputation was applied.

**Results:**

A cohort of 204 patients under DOTS, 114 men and 90 women, participated in the questionnaire survey. Total illness costs of a TB episode averaged $1053 (c. $4900 purchasing power parity, PPP), of which $292, $338 and $422 were encountered before the start of treatment, during intensive phase and in continuation phase, respectively. Costs per month were highest before the start of treatment ($145) and during intensive phase ($153) and lower during continuation phase ($95). These differences were highly significant (paired t-test, p < 0.0005 for both comparisons).

**Conclusions:**

The illness-related costs of an episode of TB exceed the per capita GDP of $1600 PPP about two-and-a-half times. Hence, these costs are catastrophic for concerned households and suggest a high risk for impoverishment. Costs are not equally spread over time, but peak in early stages of treatment, exacerbating the problem of affordability. Mitigation strategies are needed in order to control TB in Tajikistan and may include social support to the patients as well as changes in the management of TB cases. These mitigation strategies should be timed early in treatment when the cost burden is highest.

## Background

Illness often leads to substantial economic burden for the patients and their households, especially in resource-poor settings and in the absence of social support mechanisms [1,2]. Economic costs of illness at the household level-below referred to as household costs-have strong implications for issues of poverty and equitable access to health care. A review found that ill-health, especially long-lasting disease including tuberculosis (TB), puts households at high risk of impoverishment [1]. Poor households are deterred from the use of health care due to household costs, preventing the health services from reaching those most in need [3]. In the case of TB, where the main control and prevention measure consists of treating active disease [4], it must therefore be expected, that high household costs have a negative impact on TB control.

There have been many studies on cost-effectiveness of different TB control strategies, mainly investigating the costs to the health system. Important findings of these studies include the feasibility and cost-effectiveness of short-course TB chemotherapy on a predominantly ambulatory basis [5-10]. Far fewer studies measured the costs to households [11-13]. One of the first studies to comprehensively measure household costs found that the costs of an episode of TB in Thailand amounted to 20% of annual household income in the poorest third of patients and thus were devastating [12]. Other studies also found that household costs of an episode of TB were considerable-ranging from $186 to $1457 in Tanzania depending on treatment duration and around $920 in China [13,14]. A number of studies investigated reasons for delay to treatment and consequently measured only the costs up to diagnosis and registration with the TB control program [15-17]. Also when studying the economic impact on households, this strategy may be tempting, based on the rationale that TB treatment should be free of charge afterwards. However, those studies that measured costs after enrolment in TB control programs invariably found considerable costs during treatment [1,12-14]. In his review, Russell [1] found that "health expenditures tended to be lumpy, coming in peaks that intensified cost burdens over a few days or weeks". However, to our knowledge so far no study looked at the distribution of household costs of TB over time other than differentiating before diagnosis and after diagnosis.

Tajikistan is the poorest republic of the former Soviet Union with a per capita gross domestic product (GDP) of $1600 purchasing power parity (PPP, 2007 estimate) [18]. The country's infrastructure was severely damaged during the civil war from 1992 to 1998. Many villages and even whole districts are cut off in winter from electricity supply and transportation axes in this mountainous country. The economy of Tajikistan is highly informal and it has been observed that the grey economy is more important in several respects than the official economy [19]. Corruption is widespread and Transparency International ranks Tajikistan number 151 out of 180 countries in their perceived corruption index [20]. Temporary labour migration to Russia is very common, especially among young men. The health system is still very specialised, hierarchical and at least nominally comprehensive as in the Soviet Union [21]. Profound health sector and health financing reform is ongoing. Patients have to pay predefined fees for specific services above the primary care level, with different fee rates depending on referral or non-referral of the patient from primary care level. Informal payments are widespread also in the health system and out-of-pocket payments may constitute as much as 80% of total health funding [22]. In a recent survey in two districts piloting a new primary care model, almost half of the patients who had visited a family doctor reported that they had made an informal payment [23]. Involvement of the private sector in health care is very limited and virtually nonexistent outside the capital. The WHO estimate of TB incidence was 204 cases per 100,000 per year in 2006 [24], while the national TB control program estimated incidence between 160 and 180 cases per 100,000 per year. The country began DOTS implementation in 2002 and DOTS coverage reached 100% by the end of 2007. A study in ten districts of Tajikistan showed that the hospitalisation rate of TB patients was high (58%) and that a positive sputum smear result was the main predictor of hospitalisation [25]. For patients who are not hospitalised, the program foresees facility-based observation of treatment.

In most settings in the former Soviet Union anti-TB drugs are provided for free, but patients have to pay for other services including additional medicines, x-rays and laboratory services and potentially face major financial barriers [26]. In urban Ukraine, total illness-related costs of TB varied from $57 to $450 depending on the TB control strategy and on the locality [27]. These costs were often outweighed by the social benefits provided to TB patients. Overall, very little is known about illness-related costs of TB in the former Soviet Union. This study aimed at quantifying household costs of an episode of TB in Tajikistan, with special attention to the time period, when costs were incurred.

## Methods

### Study population

Only districts with an established DOTS program were considered for inclusion in the study. Two regions were excluded because they were accessible only by air at the time of the study, Badakhshan and Sughd. In close collaboration with the Republican Centre for TB control of Tajikistan, 12 districts were purposefully selected to represent different urban, rural, lowland and mountainous settings. All pulmonary TB patients at least 15 years of age who were registered for the first time (i.e. "new" patients according to WHO definition) in the 12 study districts in the period from 1^st ^December 2006 until 31^st ^March 2007 were eligible for study participation. Patients who had defaulted from treatment before the first interview were excluded. Patients who defaulted during or at the end of the intensive phase were excluded from the second interview, as they could not possibly give information on the continuation phase.

### Questionnaire and Interviews

Patients were visited in hospital or at home during the intensive phase of treatment, written informed consent was obtained and the first interview conducted. Three to four months later, the same patients were visited for a follow-up interview. The first interview covered the period before diagnosis, starting from the time point, when the patients experienced the first symptoms, and the intensive phase. The follow-up interview covered the continuation phase. The costs from the day of interview until the end of the respective phase were extrapolated, assuming a duration of four months for the continuation phase. The questionnaires were adapted from a questionnaire used for the quantification of costs of HIV/AIDS [28]. They were piloted and finalised prior to the start of data collection. They are available from the authors upon request. Completing an interview took about one hour on average. The questionnaires included detailed questions about health care seeking, about the costs incurred by patients and their households and about household assets. Household costs included medical expenditure (for drugs, medical fees and laboratory tests), non-medical expenditure (for transport, special food items, modification of housing, traditional healers, self-treatment and any other reported expenditures), and loss of income due to the inability to work. To quantify loss of income, patients were asked about the actual reduction of income that they themselves and caretakers had experienced due to absence from their usual income-generating activities for a day. This daily income reduction was then multiplied with the number of days away from work. If patients did not know the income reduction for one day, but could give an estimate for the total income reduction until the day of interview, the total estimate was registered and divided by the number of days away from work during data entry. Where income was in-kind, like sometimes in agriculture, the monetary value was estimated. Information on treatment outcome could not be obtained, as treatment was not completed at the time of the interview.

Interviewers were trained for two weeks before conducting real interviews and supervised at least twice per month through interviews conducted together with the main researcher. Specific emphasis was given to support the patients in their recall work, including the use of a calendar of locally important events, to motivate the patient to provide accurate information on costs and to ask back if reported costs were unexpectedly high or low for local context. Data were entered by the main researcher in FileMaker (version 8.0v1, FileMaker Inc, USA, 1984-2005).

### Data Analysis

All analyses were conducted in Stata IC/10.1 for Macintosh (Stata Corporation, USA, 1985-2008). Individual cost items were summed up to the categories of medical costs, non-medical costs, and lost income-for each of the three phases of treatment separately (before start of treatment, intensive phase, and continuation phase). For conversion of Tajik Somonis to international US$ PPP, we used data provided by the World Bank [29].

In order to avoid the problems associated with complete case analysis and as recommended by Manca and Palmer [30] for similar problems, we used multiple imputation to deal with missing observations, implemented in the function "ice" in Stata [31,32]. The algorithm in "ice" assumes normally distributed variables; this was assured through transformation to normal scores. The statistical comparisons of costs during the different phases were done with a paired t-test because we were mainly interested in the pair wise comparisons (n = 204 for all comparisons). To correct for the fact that we conducted three t-tests, we decreased our significance level to 1.67%. We plotted the residuals and checked visually whether the distributions were normal. In order to normalise the distribution of residuals, we used log-transformation.

We conducted a principal component analysis on 18 variables to construct a wealth index following the methodology described by Filmer and Pritchett [33]. The 18 variables included 15 household assets and three housing characteristics. To improve validity of the wealth measure, we used continuous variables, where this was possible, and we did not recode any categorical variables as dummies [34]. Subjects were allocated to five quintiles by their wealth index. To investigate the internal consistency of our measure of socio-economic status (SES), we plotted the mean of each asset variable against SES quintile and found that the asset variables were highly consistent with expectations.

The study has received ethical approval from the Ministry of Health of the Republic of Tajikistan.

## Results

In the 12 study districts, 282 eligible patients were registered for treatment during the recruitment time. Seventy-eight (28%) of them were not interviewed, because they did not consent (10 patients; 3.5%), were not at home or in hospital when visited (17; 6.0%), had died (5; 1.8%), were initial defaulters (3; 1.1%), were already in the continuation phase at the time point when visited by the research team (25; 8.9%), or the address in the registry was wrong or insufficient (18; 6.4%). The remaining 204 (72%) patients, 114 (56%) men and 90 (44%) women, were found, provided informed written consent and were interviewed. Their mean age was 31 years and they reported a median delay of 52 days until start of treatment; 122 (60%) were sputum-smear positive patients and 153 (75%) were hospitalised at some stage during the disease (table 1A). Among women, most identified themselves as housewives (58%) or farmers (12%). Among men, most reported temporary labour migration to Russia (26%) or unemployment (18%).

**Table 1:** Characteristics of study participants

**Age**	Mean	31 years
	Range	15-72 years
**Form of TB**	Pulmonary	204 (100%)
	Sputum smear positive	122 (60%)
**History of TB disease**	New cases	204 (100%)
**Delay until start of treatment**	Median patient delay	21.5 days
	Median health system delay	16 days
	Median total delay	52 days
**Hospitalisation**	Hospitalised patients	153 (75%)
	Median duration of hospitalisation	39 days
**Direct observation of treatment**	Median number of visits for treatment observation	53 visits

**Figure 1:**
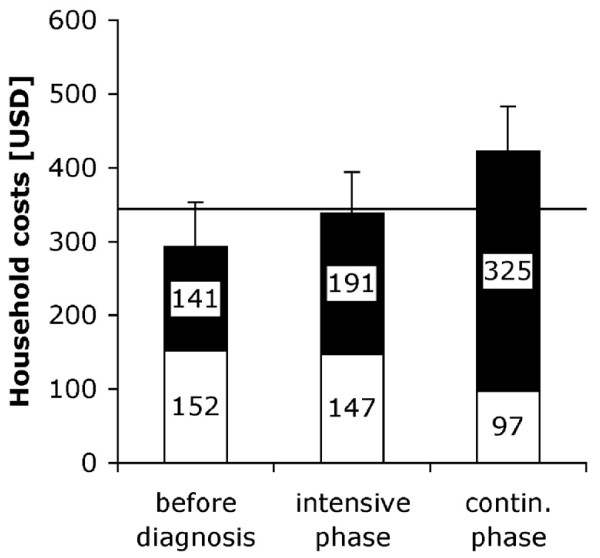
**Household costs of an episode of tuberculosis in three separate periods of treatment**. Direct costs (white), lost income (black) and standard errors of total costs are shown. The horizontal line denotes the annual per capita income [18].

The mean total household costs of an episode of TB were $1053 (± standard deviation $1601; median $378). Patients reported household costs of $292 (± 620; 54) before the start of DOTS treatment, costs of $338 (± 575; 139) during the intensive phase, and $422 (± 705; 95) during the continuation phase (figure 1). Thus about ¾ of costs were encountered after the patients were enrolled in the DOTS program. Direct costs amounted to $152 (± 219; 47), $147 (± 213; 108), and $97 (± 111; 53) for the period up to start of DOTS treatment, intensive phase, and continuation phase, respectively. Thus direct costs amounted to $396 (± 357; 282) in total over all periods and constituted 38% of total costs, the rest being lost income. Table 2A shows that direct costs most notably included costs for drugs (27% of total direct costs), transportation (25%), and special foods (29%). Drugs that were paid for mainly included vitamins and IV rehydration. The mean expenditure for the anti-TB drugs (which are officially provided for free) was very small: $0.35. Special foods included among others different kinds of meats and animal fats that were not consumed during times of good health or only in smaller amounts. Indirect costs were mainly incurred by patients themselves: 93 (79%) out of 117 patients with complete information spent some time away from usual income-generating activities. The median amount of time away from work was 135 working days. For those who worked seven days a week this would correspond to 4.5 months, for those who worked five days a week to 6.3 months. Among caretakers, 44 (36%) out of 122 cases with complete information spent a median of 24 working days away from work.

**Figure 2:**
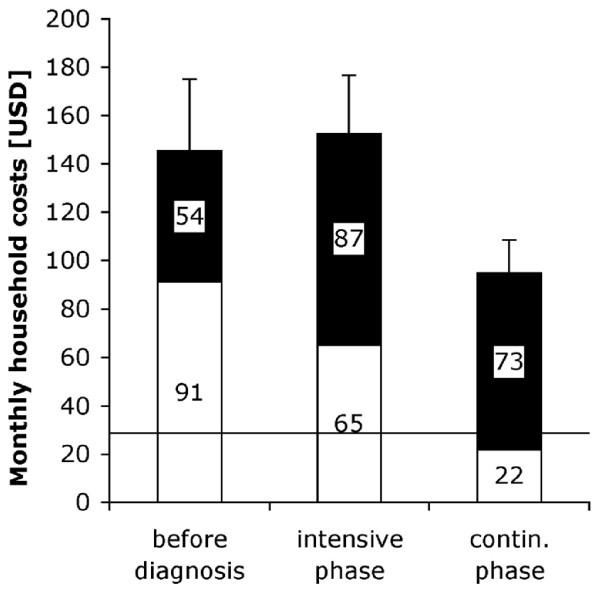
**Household costs incurred during one month during three separate periods**. Direct costs (white), lost income (black), and standard errors of the total are shown. The horizontal line denotes the monthly per capita income [18].

Household costs were not evenly spread over time. Costs incurred during one month were $145 (± 296, median 37) before start of DOTS treatment, $153 (± 256, 62) during the intensive phase, and $95 (± 159, 21) during the continuation phase (figure 2). Thus costs incurred during one month were significantly higher before start of treatment (paired t-test, p < 0.0005, n = 204) and during the intensive phase (p < 0.0005), both compared to the continuation phase. The residual plot suggested that a few observations might violate the assumption of normality. We therefore conducted a sensitivity analysis excluding these observations. Both differences were still highly significant (and in the same direction). The difference in monthly costs before start of treatment compared to the intensive phase was also significant (p = 0.001), but much smaller. While lost income was relatively constant across the three periods, direct costs per month were about 4.2 times higher before start of DOTS treatment than during the continuation phase and about three times higher during the intensive phase than during the continuation phase (p < 0.0005 for both comparisons). The difference between the period before the start of treatment and the intensive phase was not significant (p = 0.089). Thus costs were most acute during early stages of treatment. This was particularly the case for direct costs. However, patterns varied considerably for individual line items (table 3A). Costs of drugs were highest during the intensive phase and lowest before start of treatment. Costs of diagnostic and laboratory tests, and similarly costs of medical fees, were highest before start of treatment and lowest during the continuation phase. Costs of transportation were by far highest before start of treatment.

**Figure 3:**
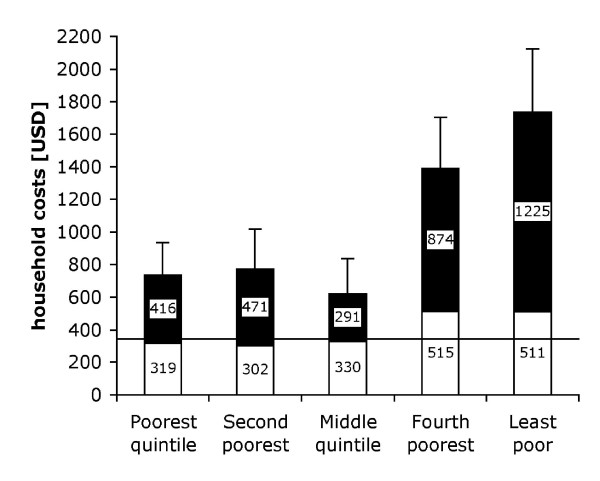
**Household costs by socio-economic quintiles**. Direct costs (white), lost income (black), and standard errors of the total are shown. The horizontal line denotes the annual per capita income [18].

Costs varied considerably by SES. Those in the lower three SES quintiles incurred less than half of the costs of the highest SES quintile on average-$735, $773 and $621 respectively versus $1735 (figure 3). This difference was mainly due to higher income loss in the higher SES quintiles, the mean income loss in the wealthiest quintile being almost three times that of the poorest. Medical direct costs were similar across all SES quintiles; non-medical costs of the wealthiest quintile were double those of the poorest ($351 versus $168).

## Discussion

The mean total household costs of a TB episode amounted to $1053, corresponding to $4894 PPP. While a considerable part of the scientific discussion on household costs has concentrated on the period before diagnosis [15-17], this study found that costs during treatment account for three quarters of total costs-in a setting with free TB drugs. Monthly costs were most acute before treatment and during the intensive phase. The total costs presented here do not yet take into account that patients may incur costs after the end of treatment; especially further loss of income for those who have lost their jobs.

The study may seem only partly representative of Tajikistan, as two regions had to be excluded. One of the regions (Badakhshan) is remote and very sparsely populated. The other one (Sughd) had only eight districts with an established DOTS program at the time of the study (versus 34 districts with DOTS in the whole country) and was only accessible by air in winter. The study is representative of an area of Tajikistan, from where 80% of all cases under DOTS came in the year up to the study.

As other studies on household costs, this study also relied on self-reported costs-it is thus not excluded that patients forgot or overestimated some of the costs [7]. By using two interviews for separate periods of treatment, this study limited recall time and recall bias. Further we tried to limit the potential problem of overestimation by training the interviewers for this situation.

The mean total household costs found in this study were even higher in terms of PPP than those found in other studies [12-14]. There may be several reasons for the high costs found in this study and these are discussed in the following. One reason may be our methodology that reduced recall bias. Further, there is a habit among TB specialists in Tajikistan to hospitalise many TB patients and to prescribe many drugs for perceived iatrogenic problems and symptomatic treatment (own data, unpubl.). Hospitalisation itself does not usually have to be paid for, and this is consistent with the fact that costs of medical fees, which include hospitalisation fees, are relatively low (cf. table 2A). However, hospitalisation may be linked to other services like the provision of additional medication. Additional drugs, including those for symptomatic treatment, have to be paid for and it seems likely that these have led to the very high costs of drugs during the intensive phase (cf. table 3A). Only a small minority of patients reported payments for anti-TB drugs. Costs of transportation were very high before the start of treatment and this may point to another reason for high costs. Thirty-one patients (15%) had developed active TB while being migrant workers in Russia. These patients encountered high transportation costs for their return to Tajikistan. An analysis of the difficulties faced by migrant workers who develop active TB in Russia is presented in a separate publication [35]. Informal payments that occur in the health system are a further potential reason for high costs. Disaggregating formal and informal payments was not possible in our study, among others because a health financing reform was ongoing during the time of the study-including formalisation of some informal payments. However, as reported above, the mean payments for anti-TB drugs were very small.

**Table 2:** Composition of household costs

Cost item	Mean in US $	Standard Deviation	Percentage of direct costs
**Indirect costs**	**657**	**1391**	**N/A**
			
**Direct costs**	**396**	**357**	**100%**
**Medical costs**	**154**	**148**	**39%**
Drug costs	107	105	27%
Costs of diagnostics and lab tests	18	19	4.5%
Costs of medical fees	29	64	7.3%
**Non-medical costs**	**242**	**286**	**61%**
Transportation	100	162	25%
Special foods	113	202	29%
Self-treatment & traditional medicine	16	41	4.0%
Other expenses	13	26	3.3%

**Table 3:** Variation of selected costs items over time [in US $]

Cost item	Before start of treatment		Intensive phase		Continuation phase	
	**Mean**	**SD§**	**Mean**	**SD§**	**Mean**	**SD§**
Drug costs	21.3	39.9	51.7	74.7	34.3	56.1
Costs of diagnostic and lab tests	7.8	16.2	7.4	8.7	2.5	3.9
Costs of medical fees	15.2	60.8	9.2	15.8	4.8	14.4
Transportation	71.2	139	8.2	12.9	20.4	26.4
Special foods	31.4	68.7	67.5	173	14.5	66.3

§SD = Standard deviation.

Household costs were approximately three times higher than the per capita GDP and thus must be considered catastrophic for affected households. As TB disproportionately affects poorer segments of society this burden may be even more catastrophic in the typical TB-affected household than average GDP values indicate. Direct costs were relatively similar for all SES quintiles, while indirect costs, which depend on income, were three times higher in the wealthiest compared to the poorest quintile. This is suggestive of a regressive cost burden at least for direct costs.

Monthly costs were especially high before enrolment in DOTS treatment and during the intensive phase. Monthly costs were significantly higher during these two periods than during the continuation phase. This pattern was very clear for direct and total costs, but less so for lost income, which was more constant over the different phases of treatment. Patients who stopped working often did so for the duration of four months and more. This suggests that they did not only stop working during the time of acute illness, but for the whole treatment. Tajik doctors often recommend them not to work during treatment and for up to two years afterwards. Seasonality of income may have led to underestimation of lost income especially before the start of treatment, because patients were enrolled into the study in the winter months, which is the season when least work is available. Consequently, the observed pattern of higher costs during early stages of treatment may even be stronger in reality. The spread of costs over time affects the ability of households to cope with these costs [1], and our findings suggest that the problem of affordability of services is most acute in early stages of treatment.

The high costs found in this study suggest that sheer poverty and inability to cope with the economic burden of treatment negatively affect treatment adherence and treatment outcomes, as has been suspected in other contexts [12,36]. Farmer et al [37] have shown that mitigation strategies including incentives for adherence can improve treatment adherence among TB patients. Donors and program managers need to implement effective mitigation strategies to enable patients to adhere to DOTS and to avoid severe impoverishment. The present study shows that these mitigation strategies must be timed early in treatment, when most of the costs are incurred. A recent study from Russia has identified types of incentives that are preferred by TB patients [38]. Additional strategies to reduce the economic burden of TB disease on households might target the high costs for drugs, which are related to the prescription patterns including common use of symptomatic treatment. Studies from Russia have shown features in the management of TB that are also found in Tajikistan, including high hospitalisation rates and widespread use of symptomatic treatment [39,40]. Furthermore similar factors influence hospitalisation in Russia and Tajikistan [25,41]. These parallels suggest that some of our results may also apply to Russia and other post-Soviet countries.

## Conclusions

During an episode of TB, patients and their households face costs of approximately $4900 PPP. Comparison with the per capita GDP of $1600 PPP shows that these costs are catastrophic for the households concerned and suggest a high risk for impoverishment. Costs are not equally spread over time, but are highest in early stages of treatment, exacerbating the problem of affordability. Free TB drugs do not lead to free treatment and non-TB drugs, transport, and loss of income are very important cost drivers for patients. Mitigation strategies are needed in order to control TB in Tajikistan and may include social support to the patients as well as changes in the management of TB cases. Mitigation strategies need to be timed early in treatment when the cost burden is highest. The provision of food supplements to TB patients may contribute to somewhat alleviate the economic burden. However, our results suggest that these should be delivered soon after a TB diagnosis. Reducing the use of non-TB drugs should be considered.

## Competing interests

The authors declare that they have no competing interests.

## Authors' contributions

RA led the study from planning to writing up. KW contributed to designing the study and to writing the manuscript. HA and SS participated in design of the study and data collection. All authors read and approved the final manuscript.

## Pre-publication history

The pre-publication history for this paper can be accessed here:

http://www.biomedcentral.com/1471-2458/10/18/prepub
